# Epithelioma of the Cervix Uteri—Removal by Prof. James P. White, M. D.

**Published:** 1880-05

**Authors:** William D. Granger


### Clinical Reports.

---

### EPITHELIOMA OF THE CERVIX UTERI—REMOVAL BY PROF. JAMES P. WHITE, M. D.

REPORTED BY WILLIAM D. GRANGER, M. D.

A few words in explanation of Dr. White's method of operating, and his ideas of epithelial cancer of the uterus, will without further comment, make clear the brief clinical history here reported. A few years ago the treatment generally taught for this disease, was the amputation of the cervix, or such part as is infra-vaginal, either by the écraseur, or the electric-cautery, or else an attempt was made to remove a more extensive tumor by the actual cautery or strong caustic preparations. These methods were applicable only in the early stages of the disease, and proved in the hands of the profession unsatisfactory. They failed to fully remove every vestige of the disease, and it soon returned with increased violence. It has been shown by microscopical study, that when the epithelial cancer is removed, and apparently healthy tissue is reached, there is in the neighboring normal structure of the uterus nests of cancerous matter, and an infiltration of cancer cells. It is necessary, therefore, not only to remove the tumor, but this diseased tissue beyond. With this end in view Dr. White has been operating for many years. Three years ago the reporter had the privilege of seeing him operate, and this principle was explained at that time. Before and since that time Dr. White has repeatedly operated for this disease, essentially in the same manner as is now reported, and with many permanent recoveries. One case, operated upon eleven years ago, has never had a return of the disease. But if the disease does show a tendency to recur, it does so as though it was a new disease, slowly at first and from a small beginning, so that by examining the patient every few months, and removing these small growths, the disease can be held in check and a prolonged, and comfortable life assured to the sufferer. It is too early now to attempt to give accurate statistics and compare them with other methods. Both time and a large number of cases are required for this purpose. But the testimony of those who have adopted this method is without exception in its favor. The principle of the method is the entire removal of every trace of cancerous disease. This is to be done though it be necessary to invade the body of the uterus, and to carry your operation to the very vicinity of the peritoneum.

Case. Mrs. B., aged sixty-four; married; has had children; has had symptoms of the disease five years; there has been pain, much loss of blood, and failure in health. An examination showed. a tumor of the cervix, including both lips of the os, extending into the vagina; the tumor on the anterior lip was the largest, it might be said to be as large as a fig; the tumor bled profusely when examined, almost hiding it, but it presented to the touch the characteristics of an epithelial growth of the so-called cauliflower variety. Patient was operated upon March 3, 1880, by Dr. White; a large, but short, round glass speculum was introduced; the soft projecting part of the tumor was broken down and scraped away with Sims' curette; this was continued until every sign of disease as far as the internal os was removed, and apparently healthy tissue was reached; the finger was introduced to search for any indurated masses of cancer in the healthy structure of the uterus, and such as were found were removed; the hemorrhage during the operation was checked by the use of pure vinegar; the removal of the mass on the anterior lip brought the operator's instrument near to the peritoneum; having removed all the cancer which could be found, the actual cautery at red heat was applied, which served two purposes: first, it stopped all bleeding; and second, it caused a slough to be formed, by which it was hoped to bring away all tissue in which there was the slightest trace of disease; a tampon of styptic iron, glycerine and cotton wool was applied, and the vagina was packed with a tampon; 1-6 gr. of morphine was given before, and 1-6 after the operation; the patient was susceptible to morphine, and although she suffered much pain, 1-24 of a grain was all that was necessary to quiet it; after a few days the tampon was removed, and the slough coming away left a healthy granulating surface, this gradually healed; on March 20th, to get greater security against a return of the cancer, fuming nitric acid was applied; this healed rapidly, and April 1st the patient was discharged, at which time the whole surface was apparently healed, and there was no discharge; the next day after the operation the temperature was 103, which was the highest point, and eight days after both pulse and temperature were normal; injections of carbolic acid were made into the vagina from the first, and nutritious food and tonics were given; the patient was directed to report every few months; the patient was much improved in her general health when discharged.

